# Moderate Activity and Fitness, Not Sedentary Time, Are Independently Associated with Cardio-Metabolic Risk in U.S. Adults Aged 18–49

**DOI:** 10.3390/ijerph120302330

**Published:** 2015-02-23

**Authors:** Jeroen H. P. M. van der Velde, Hans H. C. M. Savelberg, Nicolaas C. Schaper, Annemarie Koster

**Affiliations:** 1 Department of Human Movement Sciences, NUTRIM School for Nutrition and Translational Research in Metabolism, Faculty of Health, Medicine and Life Sciences, Maastricht University, P.O. Box 616, 6200 MD Maastricht, The Netherlands; E-Mail: hans.savelberg@maastrichtuniversity.nl; 2 Division of Endocrinology, Department of Internal Medicine, CARIM Cardiovascular Research Institute Maastricht, Maastricht University Medical Centre, P.O. Box 5800, 6202 AZ Maastricht, The Netherlands; E-Mail: n.schaper@mumc.nl; 3 Department of Social Medicine, CAPHRI School for Public Health and Primary Care, Maastricht University, P.O. Box 616, 6200 MD Maastricht, The Netherlands; E-Mail: a.koster@maastrichtuniversity.nl

**Keywords:** accelerometry, exercise, physical activity, physical fitness, sedentary lifestyle, NHANES, adult

## Abstract

This cross-sectional study is one of the first to examine and compare the independent associations of objectively measured sedentary time, moderate to vigorous physical activity (MVPA) and fitness with cardio-metabolic risk factors. We studied 543 men and women (aged 18–49 years) from the NHANES 2003–2004 survey. Sedentary time and MVPA were measured by accelerometry. Fitness was assessed with a submaximal treadmill test. Cardio-metabolic risk factors included: waist circumference (WC), BMI, blood pressure, fasting glucose, HDL- and non HDL cholesterol, triglycerides (TG), and C-reactive protein (CRP). Sedentary time, MVPA and fitness were used as predictors for the cardio-metabolic outcomes in a multiple regression analysis. Standardized regression coefficients were computed. Results show that sedentary time was associated with HDL-cholesterol (β = −0.080, *p* = 0.05) and TG (β = 0.080, *p* = 0.03). These results became non-significant after adjustment for MVPA and fitness. MVPA was associated with WC (β = −0.226), BMI (β = −0.239), TG (β = −0.108) and HDL-cholesterol (β = 0.144) (all *p* < 0.05). These results remained significant after adjustment for sedentary time and fitness. Fitness was associated with WC (β = −0.287), BMI (β = −0.266), systolic blood pressure (β = −0.159), TG (β = −0.092), and CRP (β = −0.130) (all *p* < 0.05). After adjustment for sedentary time and MVPA these results remained significant. These differences in relative importance of sedentary time, MVPA and fitness on cardio-metabolic-risk are important in the design of prevention programs. In this population, the strength of the associations between MVPA and fitness with cardio-metabolic markers appeared to be similar; both MVPA and fitness showed independent associations with cardio-metabolic risk factors. In contrast, sedentary time showed no independent associations with cardio-metabolic risk after correction for fitness and MVPA.

## 1. Introduction

Physical activity (PA) is effective in the prevention of cardiovascular disease (CVD) by improving cardio-metabolic risk factors such as hypertension, elevated LDL-cholesterol levels and increased inflammatory markers [1,2]. Consequently, current guidelines for PA advocate to spend at least 150 min per week in activities with a moderate or high intensity, known as moderate to vigorous physical activity (MVPA) [3]. Although, MVPA is usually only a small part of a person’s total PA, the majority of research on PA and health outcomes focuses on this part of the PA spectrum [4]. More recent studies have examined the association between sedentary activities and health outcomes. Sedentary activities refer to activities that do not, or only marginally increase energy expenditure above the resting level and include activities such as sitting, lying down, and watching television [5]. Higher sedentary time has been associated with increased risk of metabolic syndrome and increased levels of cardio-metabolic risk factors, independent of the amount of MVPA [6,7]. Even when PA guidelines are met, sitting for prolonged periods can still compromise metabolic health [8,9]. Nevertheless, in Western society increasingly more time is spent in sedentary activities and this trend is expected to continue given the increasing changes in private and public environments [9].

Cardiorespiratory fitness (henceforward fitness) is another important determinant of cardio-metabolic health as it is associated with a lower risk for metabolic syndrome [10] and lower CVD mortality [11]. Several studies examined the relative importance of PA and fitness as predictors for CVD or (CVD) mortality [12,13,14,15]. These studies found that fitness was more strongly related to CVD than MVPA. However these studies did not include sedentary time in their analyses; another limitation was the use of self-reported measures for PA.

In a cross-sectional study using NHANES 2003–2004 data we examined the strength of the associations between objectively measured sedentary time, MVPA and cardiorespiratory fitness with several cardio-metabolic risk factors: Body mass Index (BMI), waist circumference (WC), systolic and diastolic blood pressure (SBP and DBP), fasting glucose, HDL- and non HDL cholesterol, triglycerides (TG), and C-reactive protein (CRP) and metabolic syndrome. Obtaining insight in the relative importance of sedentary time, MVPA and fitness on these cardio-metabolic-risk factors will contribute to designing more adequate prevention programs.

## 2. Experimental Section

### 2.1. Study Population

This study used data from the National Health and Nutrition Examination Survey (NHANES) 2003–2004. NHANES is a cross-sectional study using a complex and multistage design in order to obtain a representative sample for the US population. The survey consists of a household interview by trained professionals and an examination in mobile examination centers. Data, details of methods and procedures are found on the NHANES website [16]. The study was conducted in accordance with the Declaration of Helsinki, and the protocol was approved by The National Center for Health Statistics Ethics Review Board. Written informed consent was obtained from all participants. Only participants aged from 12–49 years were eligible to perform the submaximal exercise test (n = 2809). From those, 2244 participants were selected with one or more valid days of accelerometry data. A valid day was defined as a day when the accelerometer was worn for ten hours or more. For the current analysis only participants aged ≥18 years were included (n = 1198). Further, only participants with fasting blood samples were selected (n = 583). Collection of fasting blood samples was performed in a randomly chosen subgroup. Subjects with one or more missing variables of cardio-metabolic outcomes or confounders (BMI, WC, blood pressure, glucose, cholesterol, TG, CRP, health status and smoking) were excluded (n = 27). As a final step 13 people with fasting glucose >7 mmol/L were excluded, to eliminate participants with diabetes resulting in 543 participants used in the current analysis.

### 2.2. Accelerometry

Physical activity was objectively measured using the uniaxial ActiGraph AM-7164 (ActiGraph, Ft. Walton Beach, FL, USA). This accelerometer was placed on an elastic belt and worn on the right hip. All participants were instructed to wear the accelerometer for seven consecutive days and to remove the device at bedtime and before showering, swimming and other water activities. After the wearing period the devices were returned to NHANES by mail, where the data were downloaded and the devices checked for calibration. The accelerometer monitors the intensity of vertical body movements in a one-minute time interval and records these intensities as “counts per minute” which were used to classify activity period in different intensity. Non-wear was defined as a period of >60 min with 0 counts, with allowance of 2 min of counts between 0 and 100. For our analysis, time spend in sedentary activity (<100 counts per minute) and moderate to vigorous physical activity (MVPA) (>2020 counts per minute) were calculated [17]. The proportion of time spent sedentary and time spent in MVPA was calculated by dividing the total time spent in each activity by total wear time. Hence, percent sedentary time and percent MVPA are to be interpreted as the average proportion of one day spent in this activity.

### 2.3. Cardiorespiratory Fitness

Participants performed a submaximal treadmill test in order to estimate maximal oxygen uptake (VO_2_max) as a measure for cardiorespiratory fitness; based on sex, age, BMI, and self-reported level of PA one of the eight different intensity protocols was chosen. Participants at increased risk for complications from exercise or with conditions that might affect the VO_2_max test, were excluded. The objective of every protocol was to provoke a heart rate that was approximately 75% of the age-predicted maximum by the end of the test. The exercise test included a 2-min warm-up period, two 3-minute submaximal exercise stages and a 2-min cool down period, but varied in grade and speed depending on protocol. Heart rate was monitored continuously using four electrodes connected to thorax and abdomen. The heart rate at the end of each stage was used to estimate VO_2_max. A detailed description of test procedures can be found in the NHANES Cardiovascular Fitness Procedure Manual [18].

### 2.4. Cardio-Metabolic Outcomes

WC was measured over the iliac crest. BMI (kg/m^2^) was calculated from height and body weight, which were measured with a fixed stadiometer and a Toledo digital floor scale respectively. SBP and DBP (mmHg) were measured up to four times on the upper right arm and were reported as the mean of all readings except the first, if three or four measurements were available. If participants had two valid blood pressure readings, only the last reading was used in the analysis. If participants had only one reading, that reading was used. Fasting blood samples were collected in the selected population. Glucose values were determined using the hexokinase method, with the Cobas Mira analyzer (Roche Diagnostic Systems, Inc., Montclair, NJ, USA). Serum concentrations of CRP were measured with latex-enhanced nephelometry using a Dade Behring Nephelometer II Analyzer System (Dade Behring Diagnostics, Inc., Newark, DE, USA). Serum levels total Cholesterol, HDL-Cholesterol and TG were determined with a Hitachi 704 Analyzer (Roche Diagnostics (formerly Boehringer-Mannheim Diagnostics), Indianapolis, IN, USA). Metabolic syndrome was defined as meeting three or more of the following criteria: (1) WC ≥ 102 cm for men and ≥88 cm for women; (2) serum triglyceride level of ≥1.7 mmol/L; (3) HDL cholesterol level ≤1.03 mmol/L for men and ≤1.30 mmol/L for women; (4) fasting glucose level ≥5.6 mmol/L or use of antidiabetic medications (insulin or oral agents); or (5) systolic blood pressure ≥130 mmHg and/or diastolic blood pressure ≥85 mmHg, or use of antihypertensive medications [19].

### 2.5. Covariates

Age (at screening time), sex, self-reported ethnicity (non-Hispanic white, non-Hispanic black, Mexican American and other), self-reported health status (poor to excellent on 5-point scale) and smoking status were included in the analyses as covariates. Participants with serum cotinine levels >10 ng/mL were considered current smokers [20].

### 2.6. Statistical Analysis

To obtain population representative findings, all analyses took into account the NHANES complex survey design including sample weights, stratification and clustering. The NHANES 2003–2004 fasting sample weights were used. Complex samples analyses were performed using IBM SPSS Statistics for Windows, Version 20.0. (IBM Corp., Armonk, NY, USA). Significance level was set at <0.05. Because of non-normal distribution, log transformations were used for TG, and CRP. Descriptive statistics included means and standard errors for continuous variables (age, WC, BMI, accelerometry data, fitness, blood pressure and blood sample values). Data are presented for total sample and for men and women separately to illustrate differences in the main characteristics. In further analyses data from men and women were combined. The interactions between sex and sedentary time, sex and MVPA and sex and fitness with the cardio-metabolic outcome measures were tested in all final models, all tested interactions were not statistically significant. Cardio-metabolic outcomes are presented as means with standard errors according to sex specific quartiles of the three main predictors (percent sedentary time, percent MVPA time and fitness). Differences in cardio-metabolic outcomes between quartiles of sedentary time, MVPA and fitness were tested with ANOVA.

Multiple linear regression analyses were performed with the cardio-metabolic markers as dependent variables and percent sedentary time, percent MVPA time and fitness as the independent variables. Model 1 was adjusted for age, sex, ethnicity, smoking status and self-rated health status. BMI was added in Model 2. Additionally, MVPA and fitness were added in the model of sedentary time; sedentary time and fitness were added in the model of MVPA; and sedentary time and MVPA were added in the model of fitness. Standardized regression coefficients are presented to allow for determining the relative importance of the independent variables [21]. Logistic regression analyses were performed to assess the association between quartiles of sedentary time, MVPA, and fitness with metabolic syndrome. Analyses were adjusted for age, sex, ethnicity, smoking status and self-rated health status. Additionally, MVPA and fitness were added in the model of sedentary time; sedentary time and fitness were added in the model of MVPA; and sedentary time and MVPA were added in the model of fitness.

## 3. Results

Participant characteristics are shown in Table 1. The age range of the 543 participants, of whom 55% were men, was 18–49 years, with an average of 32 years and the mean BMI was 27.1 kg/m^2^. The mean values for the blood samples were all within the normal range. Average percentage of sedentary time was 53% and average percentage of time spent in MVPA was 4.1%. Fitness (predicted VO_2_max) was 42.6 and 35.7 mL/kg/min for men and women, respectively. The correlation between sedentary time and MVPA was 0.38 and between sedentary and fitness was 0.11. A correlation of 0.29 was found between MVPA and fitness.

In Table 2 the cardio-metabolic outcomes are presented by sex-specific quartiles of sedentary time, MVPA, and fitness. Cardio-metabolic outcomes did not differ significantly between quartiles of sedentary time (all *p*-values > 0.05). More time spent in MVPA was associated with a healthier cardio-metabolic risk profile. For example, BMI was 28.5 kg/m^2^ in the lowest MVPA quartile (least active) compared with 24.9 kg/m^2^ in the highest quartile (most active). Between quartile differences were significant for all outcome measures (all *p* < 0.05), except for SBP (*p* = 0.13) and glucose (*p* = 0.09). Furthermore higher fitness was linked with advantageous values for most of the cardio-metabolic risk factors. However, differences in SBP, HDL-cholesterol, fasting glucose and TG were not significant (*p* ≥ 0.05).

**Table 1:** Participant demographics, accelerometry measures, fitness and cardio-metabolic markers.

Characteristic	Total (*N* = 543)		Men (*N* = 297)		Women (*N* = 246)	
Age (years), mean ± SE	32.19	0.57	32.02	0.91	32.37	0.73
Ethnicity, %						
non-Hispanic white	70.35%		69.18%		71.61%	
non-Hispanic black	11.04%		11.13%		10.95%	
Mexican American	11.16%		12.72%		9.46%	
Other	7.45%		6.97%		7.98%	
Waist Circumference (cm), mean ± SE	92.74	0.63	96.74	0.86	88.40	0.62
Body Mass Index (kg/m^2^), mean ± SE	27.05	0.25	27.56	0.28	26.51	0.35
Average valid wear time (min), mean ± SE	851.87	4.50	861.68	5.82	841.21	8.62
Valid days, %						
N valid days = 1	6.81%		5.72%		8.13%	
N valid days = 2	7.73%		8.42%		6.91%	
N valid days ≥ 3	85.46%		85.86%		84.96%	
Percent Sedentary Time (%), mean ± SE	53.20	0.50	51.44	0.78	55.11	0.66
Sedentary Time per day (min), mean ± SE	455.03	4.90	444.61	5.52	466.36	7.19
Percent MVPA **^a^** (%), mean ± SE	4.13	0.17	5.11	0.23	3.06	0.14
MVPA **^a^** during day (min) , mean ± SE	35.07	1.40	43.82	1.96	25.55	1.30
Fitness (mL/kg/min) , mean ± SE	39.31	0.49	42.62	0.43	35.71	0.70
SBP **^b^** (mmHg), mean ± SE	113.66	0.41	117.18	1.02	109.83	0.60
DBP **^b^** (mmHg), mean ± SE	69.27	0.56	70.65	0.68	67.76	0.79
Fasting Glucose (mmoL/L), mean ± SE	5.11	0.03	5.25	0.04	4.96	0.04
HDL-cholesterol (mmoL/L), mean ± SE	1.42	0.02	1.28	0.04	1.56	0.04
non-HDL-Cholesterol (mmoL/L), mean ± SE	3.48	0.04	3.60	0.08	3.34	0.05
Triglycerides (mmoL/L) **^c^**, mean ± SE	1.19	0.04	1.31	0.05	1.07	0.05
C-Reactive Protein (mg/L) **^c^**, mean ± SE	1.44	0.09	1.16	0.11	1.82	0.11
Metabolic syndrome, %	16.77%		19.40%		13.91%	

**^a^** MVPA: Moderate to vigorous physical activity; **^b^** SBP, DBP: Systolic and diastolic blood pressure; **^c^** back transformed from log scale.

To establish the relative importance of sedentary time, MVPA and fitness as predictors for the cardio-metabolic markers standardized regression coefficients are presented in Table 3. Sedentary time was only associated with levels of HDL-cholesterol (β = −0.080, *p* = 0.05) and TG (β = 0.080, *p* = 0.03) (Model 2). These results became non-significant after adjustment for MVPA and fitness. MVPA was negatively associated with WC (β = −0.226, *p* = 0.01), BMI (β = −0.239, *p* < 0.01) (Model 1), and TG (β = −0.108, *p* = 0.01, Model 2) and positively associated with HDL cholesterol (β = 0.144, *p* < 0.01) (Model 2). Significant associations for BMI, WC, HDL-cholesterol and TG remained after additional adjustment for sedentary time and fitness. Fitness was significantly associated with WC (β = −0.287, *p* < 0.01), BMI (β = −0.266, *p* < 0.01) (Model 1), SBP (β = −0.159, *p* = 0.03), TG (β = −0.092, *p* < 0.01), and CRP (β = −0.130, *p* < 0.01) (Model 2). These associations remained significant after adjustment for sedentary time and MVPA.

**Table 2:** Cardio-metabolic markers according to quartiles of sedentary time, MVPA, and fitness.

Independent variable	Cardio-metabolic marker	Quartile 1 (Lowest)		Quartile 2		Quartile 3		Quartile 4 (Highest)		*p* *
		Mean	SE	Mean	SE	Mean	SE	Mean	SE	
**% Sedentary Time**	Waist Circumference (cm)	92.20	1.07	92.75	1.72	93.93	1.22	91.90	2.08	0.65
	Body Mass Index (kg/m^2^)	26.77	0.41	27.48	0.76	27.29	0.44	26.62	0.71	0.72
	SBP **^a^** (mmHg)	113.35	1.25	114.57	1.13	113.09	0.88	113.69	0.95	0.78
	DBP **^a^** (mmHg)	68.98	1.00	70.12	0.96	69.29	0.91	68.59	1.18	0.76
	Fasting Glucose (mmoL/L)	5.16	0.06	5.08	0.05	5.11	0.06	5.08	0.05	0.71
	HDL-cholesterol (mmoL/L)	1.45	0.04	1.47	0.04	1.39	0.04	1.35	0.03	0.10
	non-HDL-Cholesterol (mmoL/L)	3.39	0.10	3.36	0.11	3.60	0.10	3.56	0.13	0.49
	Triglycerides (mmoL/L) **^b^**	1.09	0.06	1.15	0.06	1.21	0.07	1.32	0.08	0.27
	C-Reactive Protein (mg/L) **^b^**	1.23	0.12	1.43	0.18	1.67	0.11	1.45	0.15	0.20
**% MVPA**	Waist Circumference (cm)	95.70	2.30	94.03	1.67	93.71	1.00	87.29	1.09	<0.01
	Body Mass Index (kg/m^2^)	28.45	0.87	27.51	0.75	27.20	0.36	24.94	0.32	<0.01
	SBP **^a^** (mmHg)	116.01	0.81	112.21	1.36	113.45	0.71	112.86	1.44	0.13
	DBP **^a^** (mmHg)	70.91	0.96	68.54	0.88	70.55	0.93	67.04	0.90	<0.01
	Fasting Glucose (mmoL/L)	5.21	0.07	5.11	0.04	5.09	0.04	5.02	0.05	0.09
	HDL-cholesterol (mmoL/L)	1.35	0.06	1.32	0.04	1.40	0.05	1.60	0.05	<0.01
	non-HDL-Cholesterol (mmoL/L)	3.68	0.09	3.49	0.11	3.44	0.09	3.27	0.07	0.03
	Triglycerides (mmoL/L) **^b^**	1.33	0.07	1.28	0.06	1.14	0.06	1.01	0.05	0.01
	C-Reactive Protein (mg/L) **^b^**	2.00	0.12	1.49	0.12	1.34	0.17	1.04	0.12	<0.01
**Fitness**	Waist Circumference (cm)	98.07	1.53	92.95	2.18	91.35	1.31	87.83	1.27	<0.01
	Body Mass Index (kg/m^2^)	29.26	0.79	26.96	0.68	26.52	0.55	25.21	0.47	<0.01
	SBP **^a^** (mmHg)	116.36	1.68	113.49	1.07	113.03	0.94	111.44	1.03	0.19
	DBP **^a^** (mmHg)	70.88	1.09	70.10	0.85	69.68	1.38	65.64	0.83	<0.01
	Fasting Glucose (mmoL/L)	5.23	0.05	5.11	0.06	5.08	5.01	5.01	0.07	0.05
	HDL-cholesterol (mmoL/L)	1.42	0.05	1.38	0.05	1.41	0.05	1.45	0.06	0.83
	non-HDL-Cholesterol (mmoL/L)	3.67	0.09	3.53	0.15	3.53	0.11	3.11	0.09	0.01
	Triglycerides (mmoL/L) **^b^**	1.26	0.07	1.27	0.10	1.17	0.06	1.02	0.06	0.12
	C-Reactive Protein (mg/L) **^b^**	2.24	0.16	1.39	0.12	1.41	0.13	0.90	0.11	<0.01

**^a^** SBP, DBP: Systolic and diastolic blood pressure; **^b^** back transformed from log scale. * Between quartile differences were analyzed with one-way Anova. Quartile cut-off point % sedentary time men: 15.3%–46.2%; 46.2%–54.4%; 54.6%–61.6%; 61.8%–88.1%, women: 26.6%–46.1%; 46.3%–54.5%; 54.6%–61.8%; 61.8%–90.8%. Quartile cut-off point % MVPA men: 0.3%–2.0%; 2.1%–3.5%; 3.5%–5.9%; 5.9%–25.2%, women: 0.2%–2.1%, 2.1%–3.5%; 3.5%–5.8%; 6.1%–11.2%. Quartile cut-off point fitness (estimated VO_2_Max (mL/kg/min)) men: 26.5%–33.4%; 33.8%–38.8%; 38.9%–45.7%; 45.9%–77.7%, women: 20.9%–33.7%; 33.7%–28.9%; 39.3%–45.6%; 45.8%–78.9%.

**Table 3:** Standardized regression coefficients (β) of cardio-metabolic markers according to percent sedentary time, MVPA and fitness.

Independent variable	Cardio-metabolic marker	Model 1		Model 2		Model 2 + Sedentary Time		Model 2 + MVPA		Model 2 + Fitness		Model 2 + MVPA + Fitness	
		β	*p*	β	*p*			β	*p*	β	*p*	β	*p*
**% Sedentary Time**	Waist Circumference (cm)	−0.011	0.84					−0.074	0.21	−0.019	0.74	−0.072	0.23
	Body Mass Index (kg/m^2^)	−0.004	0.94					−0.070	0.29	−0.012	0.85	−0.067	0.31
	Systolic Blood Pressure (mmHg)	0.006	0.91	0.007	0.91			0.030	0.64	0.002	0.96	0.030	0.63
	Diastolic Blood Pressure (mmHg)	−0.006	0.90	−0.005	0.92			−0.009	0.87	−0.008	0.87	−0.009	0.87
	Fasting Glucose (mmoL/L)	−0.043	0.15	−0.043	0.13			**−0.058**	**0.04**	−0.043	0.11	**−0.058**	**0.04**
	HDL-cholesterol (mmoL/L)	−0.080	0.11	−0.080	0.05			−0.044	0.28	−0.080	0.04	−0.044	0.27
	non-HDL-Cholesterol (mmoL/L)	0.127	0.09	0.127	0.11			0.116	0.12	0.112	0.12	0.116	0.12
	Log Triglycerides (mmoL/L)	0.080	**0.04**	**0.080**	**0.03**			0.055	0.10	0.067	0.04	0.055	0.11
	Log C-Reactive Protein (mg/L)	0.055	0.07	0.055	0.06			0.047	0.14	0.055	0.07	0.047	0.13
												**Model 2 + Sedentary Time + Fitness**	
**% MVPA**	Waist Circumference (cm)	**−0.226**	**<0.01**			**−0.260**	**<0.01**			**−0.188**	**0.01**	**−0.221**	**<0.01**
	Body Mass Index (kg/m^2^)	**−0.239**	**<0.01**			**−0.271**	**<0.01**			**−0.205**	**<0.01**	**−0.236**	**<0.01**
	Systolic Blood Pressure (mmHg)	0.000	1.00	0.081	0.27	0.095	0.21			0.101	0.19	0.115	0.15
	Diastolic Blood Pressure (mmHg)	−0.048	0.29	−0.012	0.78	−0.017	0.75			−0.001	0.99	−0.005	0.92
	Fasting Glucose (mmoL/L)	**−0.080**	**0.02**	−0.050	0.07	**−0.078**	**0.01**			−0.043	0.13	**−0.070**	**0.03**
	HDL-cholesterol (mmoL/L)	**0.206**	**<0.01**	**0.144**	**<0.01**	**0.123**	**0.01**			**0.155**	**<0.01**	**0.134**	**<0.01**
	non-HDL-Cholesterol (mmoL/L)	−0.154	0.13	−0.078	0.47	−0.023	0.82			−0.068	0.54	−0.013	0.90
	Log Triglycerides (mmoL/L)	**−0.162**	**<0.01**	**−0.108**	**0.01**	**−0.082**	**0.02**			**−0.098**	**0.01**	**−0.072**	**0.03**
	Log C-Reactive Protein (mg/L)	**−0.125**	**<0.01**	−0.061	0.07	−0.038	0.27			−0.046	0.19	−0.024	0.51
												**Model 2 + Sedentary Time + MVPA**	
**Fitness**	Waist Circumference (cm)	**−0.287**	**<0.01**			**−0.287**	**<0.01**	**−0.259**	**<0.01**			**−0.258**	**<0.01**
	Body Mass Index (kg/m^2^)	**−0.266**	**<0.01**			**−0.266**	**<0.01**	**−0.236**	**<0.01**			**−0.235**	**<0.01**
	Systolic Blood Pressure (mmHg)	**−0.241**	**<0.01**	**−0.159**	**0.03**	**−0.159**	**0.03**	**−0.171**	**0.03**			**−0.171**	**0.03**
	Diastolic Blood Pressure (mmHg)	**−0.135**	**0.01**	−0.098	0.08	−0.098	0.09	−0.098	0.09			−0.098	0.09
	Fasting Glucose (mmoL/L)	**−0.099**	**0.02**	−0.071	0.07	−0.071	0.06	−0.060	0.11			−0.060	0.10
	HDL-cholesterol (mmoL/L)	0.000	0.91	−0.079	0.09	−0.079	0.07	**−0.089**	**0.03**			**−0.089**	**0.03**
	non-HDL-Cholesterol (mmoL/L)	**−0.180**	**0.02**	−0.097	0.20	−0.083	0.22	−0.086	0.26			−0.086	0.24
	Log Triglycerides (mmoL/L)	**−0.158**	**<0.01**	**−0.092**	**<0.01**	**−0.092**	**<0.01**	**−0.086**	**0.01**			**−0.086**	**<0.01**
	Log C-Reactive Protein (mg/L)	**−0.200**	**<0.01**	**−0.130**	**<0.01**	**−0.124**	**<0.01**	**−0.124**	**<0.01**			**−0.124**	**<0.01**

Model 1 is adjusted for age, sex, ethnicity, general health status and smoking. Model 2 is additionally adjusted for BMI (except for the outcome measures waist circumference and BMI). Numbers in bold indicate statistically significant results.

The strongest independent associations of MVPA and fitness were found with WC and BMI (Table 3). For these outcomes, the adjusted mean difference in WC and BMI between the most active and the least active and between the least fit and most fit were calculated. Results reveal a 5.6 cm difference in WC and 2.4 kg/m^2^ difference in BMI between the least active (lowest quartile of MVPA) and most active (highest quartile of MVPA). Similarly, a 6.3 cm difference in WC and a 2.3 kg/m^2^ difference in BMI was found between the least fit and the most fit individuals (not tabulated).

The associations between sedentary time, MPVA, and fitness with metabolic syndrome were similar to the results of the individual cardio-metabolic risk factors (not tabulated). Sedentary time was not associated with metabolic syndrome while MVPA and fitness were significantly associated with the presence of metabolic syndrome. Compared with the highest quartile of MVPA (most active); those with lower levels of MVPA had a significantly higher likelihood of metabolic syndrome in the fully adjusted model (quartile 3: odds ratio (OR): 4.24 (95% confidence interval (CI): 1.49–2.06); quartile 2: OR: 4.43 (95%CI: 1.06–18.61); quartile 1: OR: 7.96 (95%CI: 1.91–33.09). Similarly, compared to the most fit, a lower fitness was associated with an increased likelihood of metabolic syndrome (quartile 3: OR: 1.14 (95%CI: 0.42–3.08); quartile 2: OR: 1.69 (95%CI: 0.59–4.82); quartile 1: OR: 2.08 (95%CI: 1.12–3.87). In additional analyses we selected participants with three or more valid; results remained similar (data not shown).

## 4. Discussion

Using data from NHANES 2003–2004, this study is one of the first studies to evaluate the independent associations between objectively measured sedentary time, MVPA and fitness with a wide range of cardio-metabolic risk factors in a sample of US adults aged 18–49 years. Higher sedentary time was associated with lower levels of HDL cholesterol and higher levels TG but these associations were, however, not independent of MVPA and fitness. In contrast, both MVPA and fitness had an independent beneficial effect on cardio-metabolic risk. A higher level of MVPA was associated with lower WC, BMI, fasting glucose and a more favorable lipid profile (lower TG and higher HDL cholesterol), independent of sedentary time and fitness. Higher fitness was associated with lower WC, BMI and TG levels, and in addition with lower SBP, HDL and lower levels of the inflammatory marker CRP, independent of sedentary time and MVPA. Additionally, lower levels of MVPA and fitness were independently associated with a greater likelihood of metabolic syndrome.

Previous studies have shown that higher self-reported sedentary time is associated with several negative health outcomes [22,23] and more sedentary time was also associated with poor metabolic health and mortality when objective measures were used [24,25,26]. In addition, several authors examined the relationship between sedentary time and metabolic health using data from NHANES [6,7,8]. Bankoski *et al.* [6] and Genusso *et al.* [8] demonstrated in an older subpopulation (≥60 and ≥65 years respectively) that more sedentary time was associated with an increased metabolic risk independent of MVPA. Another study in a subpopulation of ≥20 years showed a linear association between sedentary time and several cardio-metabolic health outcomes, independent of MVPA [7]. However, in accordance with our results, not all studies have shown an association between higher sedentary time and poor health independent of MVPA. For example, sedentary time and metabolic risk were not associated in subjects with a mean age of 41 years [27] or in another study in adolescents after correcting for MVPA [28]. Moreover, all studies mentioned above did not include fitness in their analyses.

The lack of an independent association between sedentary time and cardio-metabolic risk in the current analyses of the NHANES data study seems in contradiction with the conclusions of Healy *et al*. [7], as these authors also used data from the same NHANES survey. A different approach in statistical analysis might explain this, as Healy *et al.* performed a trend analysis. Differences in the age of the subjects might also explain these differences (mean age 32.3 years in the present analyses *vs.* 46.5 years in the analyses by Healy *et al.*). Sedentary time may not be a risk factor for poor cardio-metabolic health in younger adults, as the effects of sedentary time possibly only become apparent in an aging population or when levels of MVPA and fitness decline below a certain threshold. More research is needed to determine the relative importance of sedentary behavior as age increases and energy expenditure in other activities decline.

In our study more MVPA and higher fitness were associated with several markers of better cardio-metabolic health and with lower likelihood of metabolic syndrome. Also earlier studies observed positive effects of MVPA and fitness on (cardio-metabolic) health [2,29,30]. In line with our results both higher levels of MVPA and fitness were independently associated with lower cardiometabolic risk [31,32,33] and inflammatory markers [33], when the effect of the other was taken into account. Other authors have compared the effects of MVPA and fitness on cardio-metabolic outcomes, and fitness seems more strongly related to cardio-metabolic outcomes and mortality than MVPA [11,14,34]. However, these results were limited by the use of self-reported PA levels and these studies did not include sedentary time in their analyses. In this study MVPA was associated with better cardio-metabolic health independent of fitness suggesting that unfit individuals could benefit from MVPA in terms of increasing metabolic health. Furthermore, fitness was associated with better cardio-metabolic health independent of MVPA, suggesting MVPA and fitness are two distinct determinants of cardio-metabolic health.

PA and fitness are related, as fitness increases with increased MVPA; other determinants of fitness include age, sex, body composition and genetic factors [35]. Fitness is considered to be a more stable trait than MVPA and decreases slowly when a person does not engage in MVPA. This could be an explanation that the observed associations of PA and fitness with metabolic health in our study do not show significant associations with the same cardio-metabolic markers. Because PA and fitness are related it could be suggested that fitness modifies the associations between PA and health. Blair *et al*. [36] and Franks *et al*. [37] have demonstrated that fitness modified the association between PA and metabolic syndrome. Especially people in the low fitness group would benefit from an increase in PA. Ekblom-Bak *et al*. [31], found that CVD risk clustered around people with low PA and low fitness. These studies all used self-reported measures of PA. In future studies, the modifying effect of fitness should preferably be studied with objective measures for PA and fitness. Because of the limited sample size in our study, we could not determine the combined effects of PA and fitness.

A strength of our study is the use of objective measures for sedentary time, MVPA and fitness, where previous studies have predominately used self-reported measures for PA. Self-reported measures of PA show limited validity and reliability [38]. The use of a sub-maximal exercise test to measure fitness is not optimal, but the estimates from sub-maximal exercise tests correlate strongly with the estimates from maximal exercise tests [39]. This report also has some limitations. Fitness was estimated using eight different protocols, intended to reach 75% of a participant’s estimated maximum heart rate by the end of stage 2. However, not all participants, especially those who had a high BMI, reached their target heart rate. This may have influenced the validity of the estimated VO_2_max. Accelerometers such as the ActiGraph used in this study, are most accurate in measuring ambulatory activities. Activities such as cycling are poorly detected and the device was not worn during water activities such as swimming. The cut-points used to define sedentary time and MVPA have been validated in younger adults and have been widely used, also in previous NHANES articles [17,40], however, they may not be appropriate for specific subgroups, for example all age-groups. Further, we did not have information about the type of physical activity as the use of accelerometry only gives us a measure of overall physical activity. A recent study shows that leisure time PA is beneficially associated with cardiovascular health, whereas occupational PA has been associated with detrimental health [41]. Because of the cross sectional design it is not possible to determine causality and longitudinal studies are needed to investigate the causal relationship of sedentary time, MVPA and fitness on metabolic health. Our analyses were adjusted for several confounders, however, residual confounding, for instance by dietary factors, may remain. Further, we did not adjust for multiple testing, consequently some of our significant findings may have emerged from type 1 errors. However, results for metabolic syndrome as an outcome were similar to the individual cardio-metabolic risk factors. Finally, NHANES is designed to provide a representative sample of the US population. However due to exclusion criteria applied during the fitness test, our study population probably does not reflect the overall population. The current analyses are based on a population without a history of, or symptoms of cardiovascular diseases, severe asthma, or other self-reported physical conditions preventing them to perform the fitness test [29], reducing the generalizability of our results. More research, in different populations using objective measures for sedentary time, PA and fitness is needed to determine the independent and combined effects of PA and fitness with health outcomes.

## 5. Conclusions

Based on our results we suggest that a combination of increasing MVPA and fitness is most beneficial to decrease cardio-metabolic risk in this adult US population. Sedentary time appears to be of minor importance for cardio-metabolic risk in this population. In the light of public health these results suggest that a relative young and healthy adult population would benefit less from interventions only directed to reduce sitting time, in terms of cardio-metabolic risk.
